# Age‐related thymic involution: Mechanisms and functional impact

**DOI:** 10.1111/acel.13671

**Published:** 2022-07-12

**Authors:** Zhanfeng Liang, Xue Dong, Zhaoqi Zhang, Qian Zhang, Yong Zhao

**Affiliations:** ^1^ State Key Laboratory of Membrane Biology, Institute of Zoology Chinese Academy of Sciences Beijing China; ^2^ University of Chinese Academy of Sciences Beijing China; ^3^ Beijing Institute for Stem Cell and Regenerative Medicine Beijing China; ^4^ Institute for Stem Cell and Regeneration Chinese Academy of Sciences Beijing China

**Keywords:** aging, T cells, thymic epithelial cells, thymic involution, thymus

## Abstract

The thymus is the primary immune organ responsible for generating self‐tolerant and immunocompetent T cells. However, the thymus gradually involutes during early life resulting in declined naïve T‐cell production, a process known as age‐related thymic involution. Thymic involution has many negative impacts on immune function including reduced pathogen resistance, high autoimmunity incidence, and attenuated tumor immunosurveillance. Age‐related thymic involution leads to a gradual reduction in thymic cellularity and thymic stromal microenvironment disruption, including loss of definite cortical‐medullary junctions, reduction of cortical thymic epithelial cells and medullary thymic epithelial cells, fibroblast expansion, and an increase in perivascular space. The compromised thymic microenvironment in aged individuals substantially disturbs thymocyte development and differentiation. Age‐related thymic involution is regulated by many transcription factors, micro RNAs, growth factors, cytokines, and other factors. In this review, we summarize the current understanding of age‐related thymic involution mechanisms and effects.

## Graphical abstract

Age‐related thymic involution has many negative impacts on immune function including reduced pathogen resistance, high autoimmunity incidence, and attenuated tumor immunosurveillance. This review summarizes the current understanding of how age impacts thymic development and function, as well as the mechanisms underlying age‐related thymic involution, particularly TEC transcriptional profile changes during thymic aging.

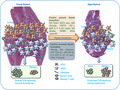

**Abbreviations**
CR: caloric restriction
cTECs: cortical thymic epithelial cells
DN: double negative
DP: double positive
EMT: epithelial–mesenchymal transition
ETPs: early T‐lineage progenitors
FGF21: fibroblast growth factor 21
GH: growth hormone
HSCs: hematopoietic stem cells
LPCs: lymphohematopoietic progenitor cells
MHCII: major histocompatibility complex class II
mTECs: medullary thymic epithelial cells
OSM: oncostatin M
PPARγ: peroxisome proliferator‐activated receptor gamma
PVS: perivascular space
SCF: stem cell factor
SP: single positive
TCR: T‐cell receptor
TECs: thymic epithelial cells
Tgfbr1: transforming growth factor beta receptor I
TRECs: T‐cell receptor excision circles

## 1 INTRODUCTION

Age‐related thymic involution is one of the most ubiquitous changes during immune system senescence. Most vertebrates experience thymic involution, which presents as a decrease in thymic epithelial cells (TECs) and accumulation of adipose tissue within the thymus. Human thymic involution is thought to begin as early as 1 year of age (Hale, 2004; Murray et al., 2003; Palmer et al., 2018). The murine thymus reaches its peak mass at 4 weeks, followed by a gradual diminution (Sutherland et al., 2005). Recently, Wu et al. examined medullary thymic epithelial cell (mTEC) transcriptomes from mice at 2, 6, and 10 weeks of age and showed that age‐related TEC degeneration occurs as early as 6 weeks after birth, as evidenced by reduced cell cycle‐related gene expression and increased inflammatory response‐related gene expression (Wu et al., 2018). Because the thymus involutes as early as 4–6 weeks in mice and 1 year in humans, ages when most other organs do not show any signs of aging, thymic involution may be an evolutionarily conserved process (Chaudhry et al., 2016). Some scientists have speculated that thymic involution has an undiscovered biological purpose and have divided thymic involution into an early phase (growth‐dependent thymic involution) and a late phase (age‐dependent thymic involution) (Aw & Palmer, 2012). The early phase (growth‐dependent thymic involution) may be due to a bioenergetic trade‐off (Boehm & Swann, 2013). In early life, it is essential to maintain high thymic activity and produce broad T‐cell receptor (TCR) diversity to protect against infections (Aw & Palmer, 2012). Once the T‐cell repertoire is established, it may be beneficial for the organism to reduce thymic activity and redistribute energy to other organs. In contrast, the late phase (age‐dependent thymic involution) may be similar to the aging process of other organs (Aw & Palmer, 2012). In this review, we mainly discuss age‐dependent thymic involution.

**FIGURE 1:**
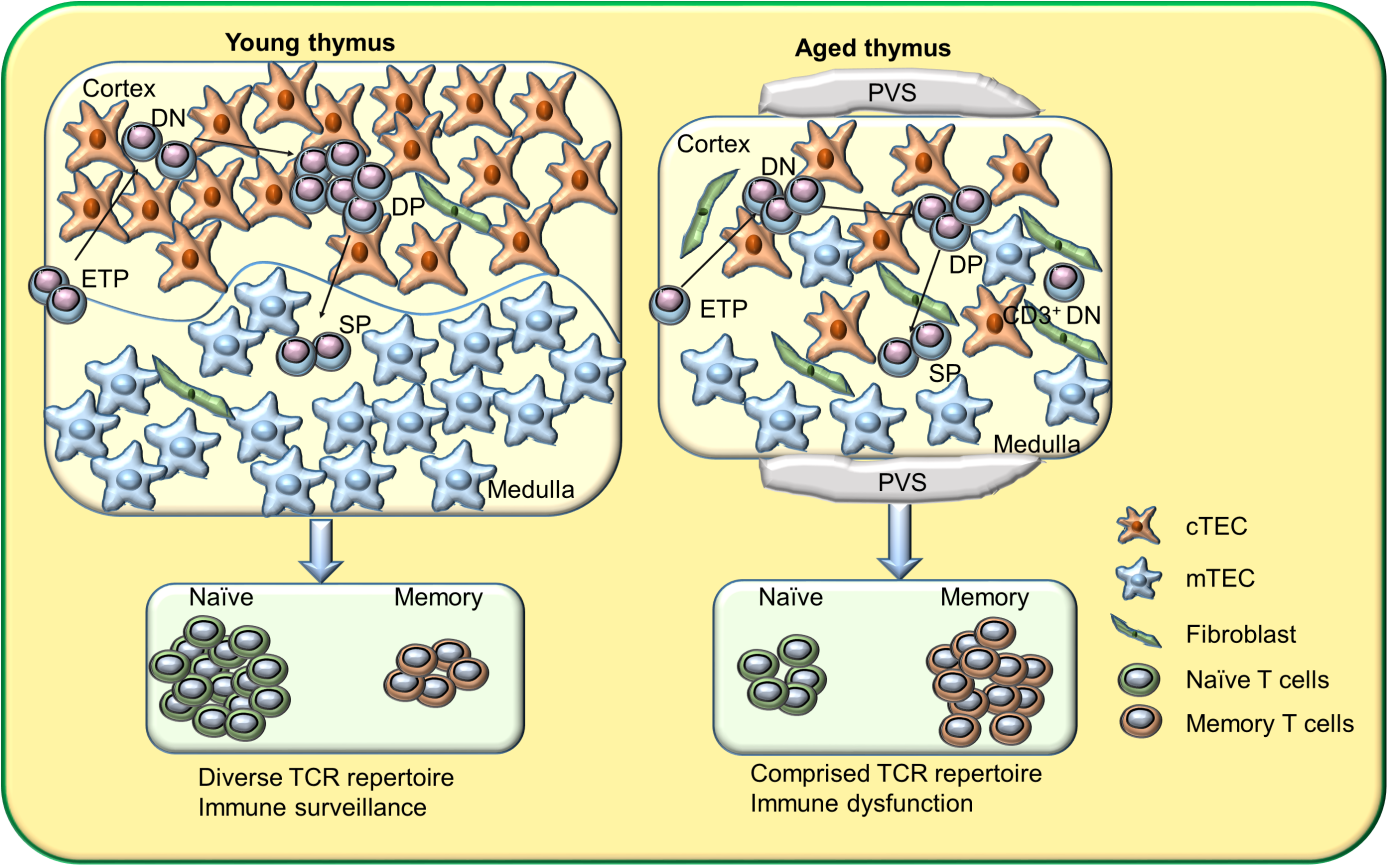
Effects of age on thymic development and function. Age‐related thymic involution leads to a gradual reduction in thymic cellularity and thymic stromal microenvironment disruption, including the loss of definite cortical‐medullary junctions, a reduction in cTECs and mTECs, fibroblast expansion, an increase in perivascular space (PVS), and more. The disrupted thymic stromal microenvironment disturbs thymocyte development causing decreased ETP and DP frequency, increased DN frequency, and abnormal CD3^+^ DN cell accumulation. The young thymus is able to produce functionally competent T cells expressing a broad TCR repertoire, whereas the aged thymus produces fewer naïve T cells with a restricted TCR repertoire

Thymic involution leads to a decline in new naïve T‐cell production and a collapse in peripheral TCR repertoire, resulting in impaired immune function (Figure 1). Thymic involution is associated with increased susceptibility to many diseases, including cancer, infection, and autoimmunity (Fahy et al., 2019; Goronzy & Weyand, 2003). It is well known that aging is associated with increased incidence of infectious diseases and neoplastic diseases, which is commonly attributed to systematic immunosenescence and a gradual accumulation of genetic mutations (Gavazzi & Krause, 2002; Pawelec, 2017). Recently, using a delicate mathematical model, Palmer et al. showed that age‐related decline in T‐cell production caused by thymic involution is a major risk factor for many cancers and infectious diseases in humans (Palmer et al., 2018). A connection between age‐related thymic demise and autoimmunity has been shown in many studies. For example, Hosaka et al. demonstrated that thymus transplantation could correct autoimmune disease in aging MRL/+ mice that exhibit dramatic thymic involution (Hosaka et al., 1996). In addition, some studies suggest that thymic aging might be involved in rheumatoid arthritis progression in humans (Goronzy & Weyand, 2003). Thus, a more complete understanding of the mechanisms and impacts of age‐related thymic involution will help us to better understand and prevent immunosenescence‐associated diseases during aging. In this review, we summarize the current knowledge on age‐related thymic involution mechanisms and effects.

## 2 THE IMPACT OF AGE ON THYMIC DEVELOPMENT AND FUNCTION

### 2.1 The impact of age on thymic stromal cells

Aged‐related thymic involution reduces thymic cellularity in mice by 50% at 16 weeks in comparison with its adult peak at 4 weeks, eventually leading to less than 5% thymic cellularity (Baran‐Gale et al., 2020; Dooley & Liston, 2012; Gray et al., 2006; Sutherland et al., 2005). In humans, thymus size reduction begins as early as 1 year of age, and it continues to decline at a rate of approximately 3% per year until middle age before slowing down to less than 1% per year (George & Ritter, 1996). Morphological analysis has shown that cortical and medullary thymic epithelial region structure becomes increasingly less reticular and less globular with age in mice, and the definite cortical‐medullary junction is also gradually lost with age (Aw et al., 2008; Baran‐Gale et al., 2020) (Figure 1). Furthermore, aging is concomitant with thymic epithelial space contraction and perivascular space (PVS) augmentation in humans (Steinmann et al., 1985) (Figure 1). The aged thymus displays obvious TEC reduction, fibroblast and adipocyte expansion, and senescent cell accumulation (Aw et al., 2008; Gray et al., 2006; Palmer, 2013). Compared with 4 week old mice, TEC cellularity is reduced by about 50% at 16 weeks and over 80% at 50 weeks (Baran‐Gale et al., 2020). In particular, the mTEC population decreases gradually with age, leading to a decline in mTEC/cortical thymic epithelial cell (cTEC) ratio in aged mice (Gray et al., 2006). Although it is widely accepted that TEC number significantly decreases with thymic aging, a recent publication indicated that thymic aging leads to the contraction of cTEC complex cell projections, but has no effect on TEC cell number in mice (Venables et al., 2019). The authors of this article speculated that the use of mechanical/enzymatic methods to isolate TECs in previous studies may have led to a gross underestimate of total TEC number, and they believed that the genetic labeling approach used in their study could overcome this (Venables et al., 2019).

Proliferation of both CD45^−^ non‐TECs stromal cells and TECs decreases dramatically with thymic aging in mice (Gray et al., 2006). Impaired TEC proliferation in aged mice was recently further demonstrated using transcriptome analysis (Cowan et al., 2019; Ki et al., 2014). Thymic aging is accompanied by a decline in TEC marker expression, including EpCAM, keratin, CD205, and Ulex Europaeus Agglutinin 1 (Aw et al., 2008). During thymic aging, the ratio of MHCII^hi^ TECs to MHCII^lo^ TECs clearly decreases, reflecting a reduced TEC antigen presentation ability in aged mice (Gray et al., 2006). However, it is notable that some recent studies have shown that the emergence of MHCII^lo^ TEC subsets during thymic development has other specific roles, such as supporting invariant NKT (iNKT) cell development in the thymus (Kozai et al., 2017; Lucas et al., 2020). Tissue‐restricted antigen expression also diminishes with age, representing a potential mechanism for age‐related increase in autoimmune diseases (Baran‐Gale et al., 2020; A. Griffith et al., 2015; Griffith et al., 2012). Aging also impairs TEC secretion ability, as demonstrated by diminished production of the thymopoietic cytokine IL‐7 in mice (Aspinall & Andrew, 2000; Ortman et al., 2002). IL‐7 administration in older mice and in the rhesus macaque increases thymic output (Aspinall et al., 2007; Pido‐Lopez et al., 2002).

Advances in bulk RNA‐seq and single‐cell RNA‐seq (scRNA‐seq) technology have allowed us to more comprehensively investigate TEC subpopulation changes and transcriptional profile changes during thymic aging. A recent scRNA‐seq study compared TEC subsets in young and old mice. Most mTECs considerably diminished and most cTECs dramatically increased in percentage upon aging, which is consistent with previous reports (Yue et al., 2019). The mTEC progenitor subsets also reduced with age; however, there was a much higher frequency of bipotent TEC progenitors in the aged thymus compared to young mice (Yue et al., 2019). It is worth noting that the mTEC and cTEC subsets in this study were divided roughly based on t‐distributed stochastic neighbor embedding analysis, and the precise nature of these subsets needs to be further elucidated experimentally. A more recent study subdivided TECs from 1, 4, 16, 32, and 52 week old mice into 9 different subtypes using scRNA‐seq analysis. In this study, the authors showed that the proportion of both perinatal cTECs and mature mTECs were significantly reduced with aging, in contrast to the proportion of mature cTECs and intertypical TECs, which increased with aging (Baran‐Gale et al., 2020). By using scRNA‐seq and lineage tracing mouse models, the authors demonstrated that intertypical TECs represent a TEC progenitor state and that aging compromises intertypical TEC differentiation into mature mTECs (Baran‐Gale et al., 2020). They further analyzed the transcriptional signatures of mature cTECs, mature mTECs, and intertypical TECs during aging and found that an inflamm‐aging transcriptional signature was restricted to mature cTECs and mature mTECs, rather than intertypical TECs (Baran‐Gale et al., 2020).

By comparing thymic stromal cell population transcriptomes from 1‐, 3‐, and 6‐month‐old mice, Ki et al. found that the expression of E2F3 transcriptional targets and cell cycle‐associated genes decreased with early thymic aging in cTECs and mTECs (Ki et al., 2014). A similar study showed that the decline in E2F3 transcriptional targets and cell cycle‐associated genes occurs as early as 6 weeks in mice (Wu et al., 2018). E2F3 is a transcription factor that regulates cell proliferation and many cell cycle‐associated genes (Humbert et al., 2000); thus, reduced E2F3 activity results in decreased TEC cell‐cycle progression in aged mice. Cell cycle‐related gene downregulation during thymic aging was further confirmed by another study that showed a decline in myc targets and ribosomal genes with thymic aging in mice (Cowan et al., 2019). By using a FoxN1MycTg mouse model, in which myc is overexpressed in TECs, the authors further demonstrated that myc mainly promotes ribosomal gene expression in TECs, which are distinct from cyclin D1 regulated genes (Cowan et al., 2019). These bulk RNA‐seq and scRNA‐seq results provide an overview of TEC transcriptional and cell subset changes during thymic aging. Some representative cTEC and mTEC transcripts that are downregulated during aging are summarized in Table 1.

**TABLE 1:** The expression levels of the representative downregulated genes in mTECs and cTECs during aging

Cell types	Genes		Average expression level (TPM)		
			Newborn	Adult	Aged
mTECs	Cell cycle‐related genes or E2F3 targets	Ccna2	75.87	63.14	41.00
		Ccnb1	93.8	86.26	60.38
		Ccnb2	73.15	56.97	41.63
		Cdk1	96.96	78.58	57.27
		Cdkn2d	38.29	34.65	19.96
	Ribosomal genes	Rpl23a	1224.37	1002.92	760.76
		Rpl10a	361.87	330.44	242.68
		Rps24	601.92	545.33	344.34
		Rps29	1714.98	1339.24	919.09
		Rpl9	1313.21	1105.58	726.28
cTECs	Cell cycle‐related genes or E2F3 targets	Ccna2	53.09	41.22	5.92
		Ccnb1	52.71	53.66	2.13
		Ccnb2	38.14	80.92	8.57
		Cdk1	64.35	21.59	2.75
		Cdkn2d	39.73	45.84	33.06
	Ribosomal genes	Rpl23a	1267.49	827.83	626.10
		Rpl10a	531.99	326.59	210.05
		Rps24	728.18	398.52	180.39
		Rps29	1696.40	881.87	426.52
		Rpl9	2077.67	986.22	662.24

*Note*: According to the RNA‐seq data of Cowan et al. (2019).

In addition to TECs, aging also affects other stromal cells in the thymus. Thymic aging coincides with adipocyte accumulation around the thymus, and the increase in adipose tissue may inhibit thymic function through adipocytokine secretion (Dixit, 2010). Fibroblast percentage also increases in the aging thymus (Figure 1) in species including mice, humans, and fish, suggesting that this may be a conserved feature (Bertho et al., 1997; Gray et al., 2006; Torroba & Zapata, 2003). Recently, a thymic stromal cell transcriptome analysis revealed that proinflammatory gene expression increased with aging in mouse thymic dendritic cells, which in turn may accelerate thymic aging (Ki et al., 2014). Another interesting study demonstrated that thymic B cell function is also impaired with aging in mice; the authors showed that Aire and Aire‐dependent tissue‐restricted antigen expression decline in aging thymic B cells (Cepeda et al., 2018). Thus, aging impairs many cell subsets in the thymic microenvironment.

### 2.2 Thymocyte development in the aged thymus

In addition to thymic stromal cells, thymocyte development is also drastically disturbed during thymic aging. Some studies have shown that hematopoietic stem cells (HSCs) of aged mice display an increased bias toward myeloid differentiation concomitant with a diminished lymphoid lineage differentiation ability (Beerman et al., 2010). HSC abnormalities in aged mice may affect the seeding of early T‐lineage progenitors (ETPs) within the thymus. Indeed, ETP frequency declines with aging, and their potential ability to reconstitute the thymus is also reduced (Min et al., 2004, 2005). ETPs from young mice are able to differentiate into all stages of thymocytes when seeded into thymic lobes; in contrast, this differentiation ability is impaired in ETPs from aged mice (Min et al., 2004). However, the effect of aging on HSCs and ETPs is controversial. Zhu et al. established an elegant mouse model in which they transplanted a fetal thymus into the kidney capsule of aged mice, thus providing a young thymic microenvironment for aged lymphohematopoietic progenitor cells (LPCs) (Zhu et al., 2007). Using this model, they demonstrated that the LPCs derived from aged mice and young mice have similar abilities to differentiate into ETPs and subsequent thymocyte subpopulations when transplanted into the young thymic microenvironment, indicating that LPCs do not have a defect synchronized with age‐related thymic involution (Zhu et al., 2007). Another study showed that the ETP defects in aged thymi are mainly due to changes in thymic epithelial architecture, including the poorly defined cortico‐medullary junction and reduced medulla cellularity, rather than ETP‐intrinsic defects (Gui et al., 2007). Thus, although aging may have some effects on HSCs and ETPs, the impairment of ETPs and subsequent thymocyte subpopulations in aged mice can be mainly attributed to thymic microenvironment disruption.

ETPs subsequently differentiate into double negative (DN) (CD4^−^CD8^−^) subpopulations that include DN1 (CD44^+^CD25^−^), DN2 (CD44^+^CD25^+^), DN3 (CD44^−^CD25^+^), and DN4 (CD44^−^CD25^−^) (Liang, Zhang, Dong, et al., 2021; Luan et al., 2019). The DN subsets subsequently become double positive (DP) (CD4^+^CD8^+^) cells that further differentiate into CD4 or CD8 single positive (SP) T cells through the process of positive and negative selection (Germain, 2002). Although both DN and DP population cell numbers are significantly reduced with aging, DN subset frequency increases 2–3 times in aged (24–27 months old) mice compared with young (2–3 months old) mice, whereas the percentage of DP (CD4^+^CD8^+^) subpopulations significantly diminishes with age (Thoman, 1995). Among DN subsets, there is a considerable reduction in DN2 and DN3 subset cell numbers with thymic aging in mice (Aspinall, 1997). Additionally, thymic aging is concomitant with the abnormal accumulation of CD3^+^ DN cells within the thymus (Aw et al., 2009, 2010). Aging also interferes with later stages of thymocyte development. DP and SP thymocytes in aged mice display deregulated CD3 expression, which may lead to attenuated TCR‐dependent stimulation (Aw et al., 2009). Indeed, thymocytes from older mice exhibit an impaired mitogen response ability, which is manifested by a failure to upregulate the activation marker CD69 and proliferate (Aw et al., 2010; Djikic et al., 2014). Consistent with impaired thymocyte differentiation in the aged thymus, T‐cell receptor excision circles (TRECs) within the thymus also significantly decline with aging in mice and humans (Ortman et al., 2002; Palmer et al., 2018). Thus, aging impairs multiple thymocyte developmental stages.

### 2.3 Thymic involution effects on thymic output

Mature CD4 SP and CD8 SP thymocytes are exported to the periphery where they play a role in immunological surveillance (Liang, Zhang, Zhang, et al., 2021; Zhang et al., 2021). Age‐related thymic involution causes an obvious reduction in the thymic output of naïve T cells and subsequently decreases peripheral T‐cell diversity (Chaudhry et al., 2016; Cowan et al., 2020). Diminished thymic production of naïve T cells leads to homeostatic expansion of existing T cells, resulting in memory T‐cell augmentation (Surh & Sprent, 2000). Although it is well accepted that the thymic output of peripheral naïve T cells progressively declines with aging in mice (den Braber et al., 2012), in humans, the relationship of thymic involution to peripheral naïve T‐cell maintenance is a matter of debate. Many studies using TRECs as a measurement of thymic output demonstrate that peripheral naïve T‐cell thymic output declines with aging in humans (Fagnoni et al., 2000; Ferrando‐Martinez et al., 2011; Mitchell et al., 2010; Naylor et al., 2005). However, Braber et al. showed that adult human peripheral naïve T‐cell pool maintenance occurs almost exclusively through cell proliferation, rather than thymic output (den Braber et al., 2012). Thus, the contribution of thymic output to naïve T‐cell pool maintenance in adults needs further investigation.

Aging also interferes with naïve T‐cell properties and functions (Srinivasan et al., 2021). Naïve T cells from aged mice express elevated levels of senescence markers and display reduced proliferation ability upon antigen stimulation (Akbar & Henson, 2011; Chaudhry et al., 2016). Chemokine receptor expression is also altered in CD4^+^ T cells of aged mice, exhibiting a deregulation of CCR1, 7, and 8 and CXCR2, 4, and 5, which may impair their migration ability (Mo et al., 2003). The reduced number of naïve T cells together with the disrupted function of naïve T cells during aging leads to impaired immunological surveillance ability in aged organisms.

## 3 AGE‐RELATED THYMIC INVOLUTION MECHANISMS

### 3.1 Thymic stromal cell alterations lead to thymic involution

Although the T‐lineage differentiation potential of HSCs and ETPs is partially compromised in aged mice compared with young mice (Min et al., 2004; Zediak et al., 2007), increasing evidence suggests that thymic involution is mainly caused by age‐related thymic stromal cell degeneration, particularly TEC degeneration (Chen et al., 2009; A. V. Griffith et al., 2015; Gui et al., 2012; Zhu et al., 2007). For example, a global transcriptome analysis of thymic stromal cells and lymphocytes revealed that mouse thymic stromal cells, in contrast to lymphocytes, are deficient in catalase (A. V. Griffith et al., 2015). This results in elevated H_2_O_2_ levels and stromal cell oxidative damage, which subsequently leads to thymic atrophy. The authors further showed that thymic atrophy could be ameliorated by genetic and biochemical restoration of antioxidant activity (A. V. Griffith et al., 2015). Similar to this study, another publication revealed that the thymi of human Down syndrome patients exhibited premature senescence, and TECs from Down syndrome patients showed increased oxidative stress (Marcovecchio et al., 2021). Using a genome‐wide computational approach, another group showed that age‐associated thymic degeneration is primarily a stromal cell function change (Griffith et al., 2012). Many studies support the pivotal role of thymic stroma in thymic aging. Mackall et al. showed that lethally irradiated older mice exhibit impaired thymopoiesis compared with lethally irradiated young mice after both were injected with young bone marrow (Mackall et al., 1998). A similar experiment showed that intrathymic injection of young ETPs failed to restore normal thymopoiesis in older mice but did so in young mice (Zhu et al., 2007). In contrast, the same study showed that fetal thymi transplants into the kidney capsules of young or old mice had similar thymopoiesis (Zhu et al., 2007). Overall, these findings suggest that the thymic stroma is a key factor in regulating age‐related thymic involution. Likely, the durable identity of the thymus is established by its stromal components because developing thymocytes are only transiently present in the thymus (Petrie & Zuniga‐Pflucker, 2007).

### 3.2 Molecular regulation of thymic involution

**FIGURE 2:**
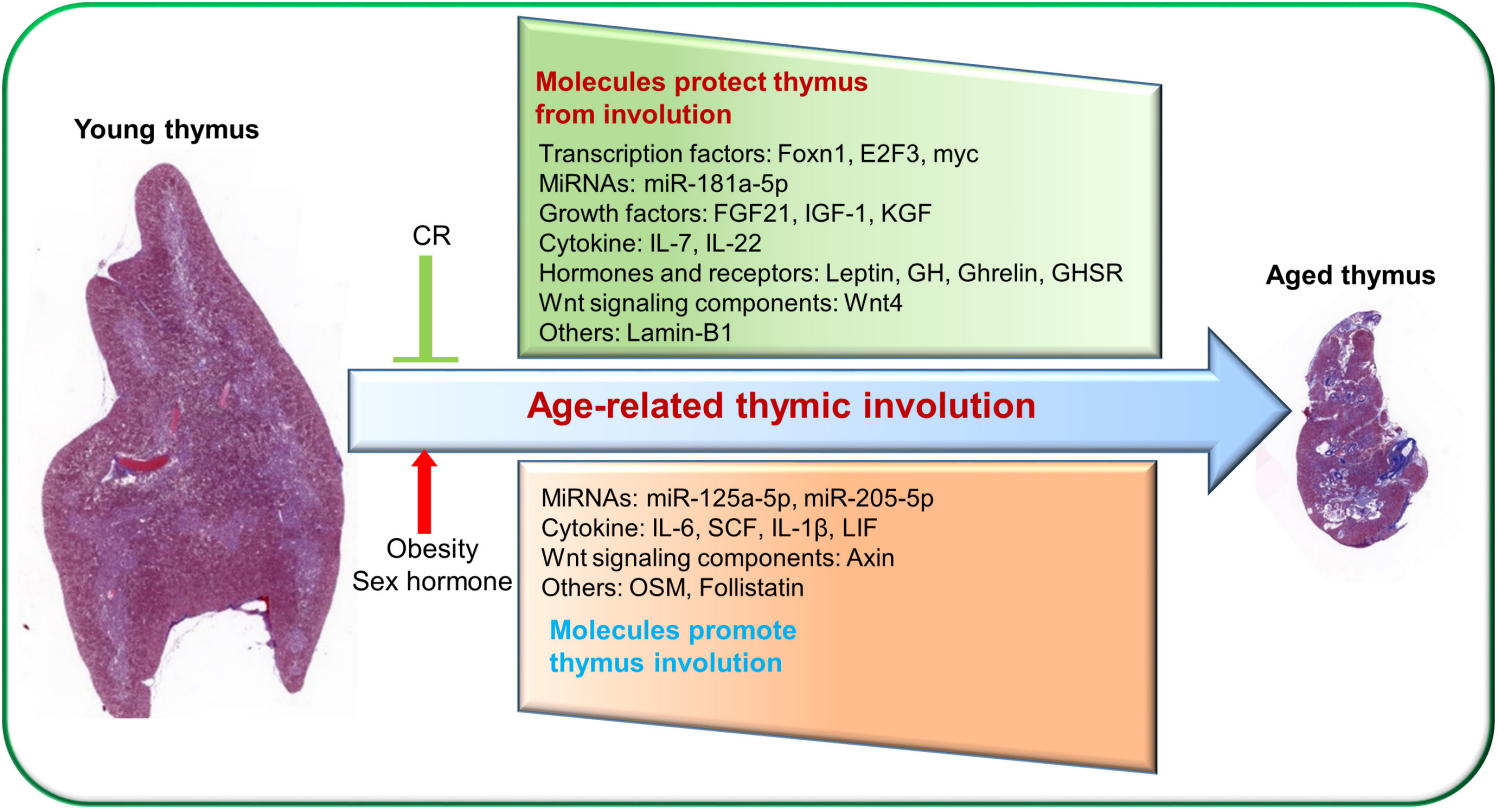
Age‐related thymic involution mechanisms. Both positive and negative regulators of thymic involution have been identified. Positive regulators include Foxn1, E2F3, myc, Wnt4, FGF21, KGF, IL‐7, IL‐22, miR‐181a‐5p, Lamin‐B1, Leptin, GH, IGF‐1, Ghrelin, and GHSR, which exhibit reduced activity with age. Negative regulators include Axin, LIF, OSM, IL‐6, SCF, IL‐1β, miR‐125a‐5p, miR‐205‐5p, and follistatin, which exhibit increased activity with age. In addition, CR can attenuate age‐related thymic involution, while obesity and sex hormones exacerbate age‐related thymic involution

Foxn1 is essential for embryonic thymic organogenesis and TEC maintenance in adults (Zuklys et al., 2016), and emerging evidence suggests that Foxn1 also plays a critical role in preventing age‐related thymic involution (Abramson & Anderson, 2017). Foxn1 expression progressively declines with aging (Figure 2), and Foxn1 overexpression ameliorates age‐related thymic deterioration, indicating that Foxn1 is a pivotal regulator of thymic aging (Bredenkamp et al., 2014; Chen et al., 2009; O'Neill et al., 2016; Rode et al., 2015; Zook et al., 2011). Sun et al. generated a loxP‐floxed‐Foxn1 mouse model carrying a ubiquitous CreERT transgene with a low level of spontaneous activation leading to a gradual loss of Foxn1 expression with age (Sun et al., 2010). By examining this mouse model's phenotype at different ages, Sun et al. demonstrated that gradual Foxn1 loss with age substantially accelerates age‐related thymic involution (Sun et al., 2010). In contrast, Foxn1 overexpression restores most of the changes caused by thymic involution in old mice, including thymic mass enlargement, increased ETP frequency, elevated EpCAM^+^MHCII^+^ TEC cell number, and CD4^+^ and CD8^+^ naïve compartment expansion in the spleen (Bredenkamp et al., 2014; Zook et al., 2011). Moreover, a recent study showed that engrafting Foxn1‐reprogrammed embryonic fibroblasts could rejuvenate aged thymic architecture and function in both male and female mice (Oh et al., 2020). Collectively, these studies demonstrate a crucial role for Foxn1 in regulating age‐associated thymic degeneration.

The Wnt signaling pathway attenuates with aging in the thymus (Ferrando‐Martinez et al., 2015; Kvell et al., 2010; Yang, Youm, Sun, et al., 2009) (Figure 2). Ferrando‐Martínez et al. found that the nonadipocytic component of the human thymus expresses higher levels of Wnt pathway inhibitors in the elderly than in the young, thus attenuating the Wnt pathway (Ferrando‐Martinez et al., 2015). Using thymic stromal cell transcriptome analysis, Griffith et al. revealed that Wnt signaling deregulation is the most significant hallmark of thymic degeneration (Griffith et al., 2012). A previous study illustrated that the expression of Axin, a Wnt inhibitor, on mTECs and fibroblasts increases with aging in humans and mice, and Axin knockdown by RNA interference ameliorates age‐related thymic degeneration (Yang, Youm, Sun, et al., 2009). Another study showed that Wnt pathway reduction during aging may involve the epithelial–mesenchymal transition (EMT) process in mice, which we will discuss further below (Kvell et al., 2010).

Growth factors and cytokines also play critical roles in age‐related thymic involution. Prolongevity ketogenic hormone fibroblast growth factor 21 (FGF21) expression gradually declines in the thymus with age, and loss of FGF21 function in middle‐aged mice accelerates age‐dependent thymic deterioration (Figure 2), suggesting that FGF21 expression could protect against age‐related thymic involution (Youm et al., 2016). Leukemia inhibitory factor (LIF), oncostatin M (OSM), IL‐6, and stem cell factor (SCF) expression levels all increase with age in mice (Figure 2), and this elevated expression is associated with thymic involution (Sempowski et al., 2000). Studies have shown that these cytokines may originate from adipocytes or TECs (Dooley & Liston, 2012; Ventevogel & Sempowski, 2013). Another proinflammatory cytokine, IL‐1β, is mainly expressed by macrophages in the thymus (Figure 2), and its increased expression levels also lead to thymic involution (Dixit, 2012; Finn et al., 2018; Guarda et al., 2011). Consistent with this, IL‐1β receptor is primarily expressed in TECs, and ablation of Nlrap3 and Asc, which are required for IL‐1β activation, protect against age‐related thymic demise and immunosenescence in mice (Youm et al., 2012). In contrast, some cytokines and growth factors play positive roles in preventing age‐associated thymic degeneration. Studies have shown that IL‐7, IL‐22, and keratinocyte growth factor administration reverse age‐induced thymic involution in humans and mice (Ventevogel & Sempowski, 2013).

MicroRNAs (miRNAs) have been implicated in the aging process in many organisms, and the role of miRNAs in age‐associated thymic deterioration was recently investigated. Guo et al. compared various miRNA expression levels in TECs from 2‐month‐old and 20‐month‐old mice and identified many differentially expressed miRNAs (Guo et al., 2013). Whole thymus miRNA expression has also been examined during aging. Compared with thymi from 1‐month‐old mice, 50 and 81 miRNAs were differentially expressed in thymi from 10‐month‐old and 19‐month‐old mice, respectively (Ye et al., 2014). Among these differentially expressed miRNAs, miR‐181a‐5p and miR‐125a‐5p, which were downregulated and upregulated during aging, respectively, have been studied further. Guo et al. revealed that miR‐181a‐5p expression decreased in TECs of 10‐ to 19‐month‐old mice compared to 1‐month‐old mice, and miR‐181a‐5p promoted mTEC proliferation by targeting transforming growth factor beta receptor I (Tgfbr1), which exhibits increased expression with aging. This suggests that miR‐181a‐5p could prevent age‐related thymic demise by interfering with TGFβ signaling that could negatively regulate the development of mTECs and promote thymic involution (D. G. Guo et al., 2016; Hauri‐Hohl et al., 2008, 2014; Xu et al., 2018). In contrast to miR‐181a‐5p, Xu et al. revealed that miR‐125a‐5p expression increased in TECs of aged mice compared with TECs of young mice. They found that miR‐125a‐5p suppressed Foxn1 expression, which may underlie its role in promoting age‐related thymic involution (Xu et al., 2018; Xu, Sizova, et al., 2017). A more recent study showed that miR‐205‐5p expression in TECs markedly increased with aging in mice, and miR‐205‐5p promoted age‐associated thymic involution by inhibiting TEC proliferation (Gong et al., 2020). Another study compared differentially expressed miRNAs in the thymi of 10‐month‐old newborn babies and 70‐year‐old humans and showed that 106 miRNAs were significantly changed in elderly thymi (Ferrando‐Martinez et al., 2015). Furthermore, some of the altered miRNAs in this study, such as miR‐25, miR‐134, and miR‐7f, could modulate the Wnt pathway (Ferrando‐Martinez et al., 2015). In addition to age‐related thymic involution, microRNAs also regulate stress‐induced thymic involution. Papadopoulou et al. showed that miR‐29a could prevent pathogen‐associated thymic involution via targeting the IFN‐α receptor in TECs, and Hoover et al. revealed that miR‐205 expression in TECs could maintain thymopoiesis following inflammatory perturbations in mice (Hoover et al., 2016; Papadopoulou et al., 2012). Thus targeting miRNAs may be a potential strategy to rejuvenate age‐induced diminished thymic function (Xu, Zhang, et al., 2017).

Recently, some new thymic involution regulators have been identified. Lamin‐B1 is a cellular architectural protein that has recently been shown to play a critical role in preventing thymic aging in mice (Yue et al., 2019). Yue et al. demonstrated that the increased proinflammatory cytokines produced by thymic myeloid immune cells during aging diminishes Lamin‐B1 expression in TECs and promotes cell senescence, which subsequently induces age‐related thymic involution (Yue et al., 2019) (Figure 2). Other recent studies have shown that imbalances in follistatin, activin A, and BMP4 signaling drive thymic involution in mice (Lepletier et al., 2019), while liver X receptors, a class of nuclear receptors that sense intracellular oxysterols and cholesterol biosynthetic pathway intermediates, may protect against premature thymic involution in mice (Chan et al., 2020). Additionally, results from our lab showed that TEC‐specific deletion of tuberous sclerosis complex 1 (Tsc1), a negative regulator of mTOR activity (Liang, Zhang, Zhang, et al., 2021), also accelerates thymic involution in mice (unpublished data). Interestingly, sirtuin 6 (Sirt6) is a chromatin deacylase that has been implicated as a key factor in aging (Chang et al., 2020); however, our recent publication showed that Sirt6 deficiency in TECs has no obvious effects on thymic aging in mice (Zhang et al., 2021).

### 3.3 Sex hormones in thymic involution

Steroid hormone levels change dramatically with aging, and steroid hormones play a critical role in promoting age‐related thymic involution (Gui et al., 2012). The role of sex hormones in thymic involution was first reported in 1904 in a study that found that castrated cattle had enlarged thymi (Henderson, 1904). Additionally, the fact that the thymus degenerates most rapidly after puberty, when steroid hormone production reaches its peak, further supports the role of steroid hormones in thymic involution (Abramson & Anderson, 2017). Thymic involution is also more rapid in males than in females (Gui et al., 2012; Hun et al., 2020), implying that androgens may have a more dramatic impact on thymic involution. Although both TECs and thymocytes express androgen receptors (Olsen et al., 2001), androgen‐mediated thymic involution is caused by direct impact on TECs rather than thymocytes because TEC‐specific (but not thymocyte‐specific) androgen receptor deletion leads to androgen‐mediated thymic involution resistant in mice (Olsen et al., 2001). More recently, a comprehensive transcriptome analysis showed that sexual dimorphism significantly affects cTECs (Dumont‐Lagace et al., 2015). cTECs from male mice display low proliferation rates, and androgen‐dependent signaling represses the expression of genes involved in cTEC development and function, such as Foxn1, Dll4, Psmb11, and Ctsl (Dumont‐Lagace et al., 2015). Consistently, another study demonstrated that sex steroid blockade could increase Dll4 expression and its downstream targets on cTECs in mice, which further promotes thymopoiesis by modulating Notch signaling (Velardi et al., 2014). Notably, although castration is an effective way to regenerate the aged thymus, the thymic regrowth induced by castration is transient (Griffith et al., 2012).

Pregnancy also causes thymic involution, mainly mediated by progesterone (Clarke & Kendall, 1989). Studies have shown that progesterone receptor expression in thymic stromal cells is required for thymic involution during pregnancy in mice (Tibbetts et al., 1999). Interestingly, thymic involution during pregnancy may be essential for normal fertility (Tibbetts et al., 1999). A recent study uncovered that RANK expression in TECs promoted sex hormone‐mediated thymic involution and natural regulatory T‐cell development during pregnancy, which is critical for successful pregnancy and prevention of gestational diabetes (Paolino et al., 2021).

### 3.4 Metabolic regulation of thymic involution

Caloric restriction (CR) has long been known to play a critical role in increasing life span. Recently, CR was also shown to be effective at preventing age‐related thymic involution. Yang et al. showed that CR could inhibit thymic adipogenesis and reduce age‐related thymic involution in mice (Yang, Youm, Vandanmagsar, et al., 2009). Another study conducted on nonhuman primates obtained similar results; the authors showed that long‐term CR effectively improves naïve T‐cell production and preserves T‐cell receptor repertoire diversity (Messaoudi et al., 2006). Thymus transcriptome analysis in short‐term CR mice showed that CR altered catalytic activity and metabolic processes (Omeroglu Ulu et al., 2018). Short‐term CR also altered the expression of leptin, ghrelin, Igf1, and adiponectin, some of which were reported to be associated with age‐related thymic involution (Omeroglu Ulu et al., 2018).

Obesity increases the risk of infections and cancer, which may be partly ascribed to obesity's negative impact on thymic involution. High‐fat diet fed mice display disrupted thymic structure, including a reduced medullary region and an absence of the cortico‐medullary junction (Gulvady et al., 2013). Diet‐induced obesity also leads to thymocyte apoptosis, reduces thymic output, and compromises TCR repertoire diversity in mice (Yang, Youm, Vandanmagsar, et al., 2009). Progressive adiposity in middle‐aged humans also decreases thymic output (Yang, Youm, Vandanmagsar, et al., 2009). Resveratrol, a phytoalexin produced from plants, has been shown to have the potential to inhibit obesity‐induced thymic involution (Gulvady et al., 2013; Wei et al., 2020). Leptin is a potent adipokine that is responsible for sensing a positive energy balance state and reducing food intake (Friedman & Halaas, 1998). Leptin (ob/ob mouse)‐ and leptin receptor (db/db mouse)‐deficient mice display severe obesity that subsequently causes significant thymic involution (Dixit, 2012; Howard et al., 1999). Leptin administration rescues this accelerated thymic involution in ob/ob mice (Howard et al., 1999). Another study showed that leptin receptor is mainly expressed in the medullary region of the thymus (Gruver et al., 2009). Consistent with results in mice, human patients with loss‐of‐function leptin and leptin receptor mutations also display T‐cell functional defects that could be partially reversed by recombinant leptin administration (Farooqi et al., 2002, 2007). Furthermore, naïve CD8 T‐cell maintenance in nonagenarians has been shown to be associated with high leptin levels (Chen et al., 2010).

Growth hormone (GH) and its proximal mediator, IGF‐1, play critical roles in preventing age‐associated thymic involution. Indeed, GH removal by hypophysectomy leads to thymic atrophy in mice and humans (Napolitano et al., 2008; Savino et al., 2002). Circulating GH levels decline with aging, and GH administration partially ameliorates age‐related thymic involution in mice (Taub et al., 2010). Randomized clinical studies in middle‐aged HIV patients showed that GH treatment increases thymic mass and elevates TRECs in peripheral T cells (Napolitano et al., 2008). Similarly, exogenous administration of IGF‐1 enhances thymopoiesis mainly through TEC expansion in mice (Chu et al., 2008). Furthermore, subcutaneous transplantation of GH_3_ pituitary adenoma cells, which secrete growth hormone, reverses thymic aging in rats (Kelley et al., 1986). A recent clinical trial showed that recombinant human GH administration combined with dehydroepiandrosterone and metformin could promote thymic regeneration and increase protective immunological changes (Fahy et al., 2019). However, GH application in clinical practice needs to be carefully considered due to its significant side effects (Taub et al., 2010). Ghrelin is a stomach hormone that can induce strong GH‐releasing activity through binding to its receptor‐specific 7‐transmembrane GH secretagogue receptor (GHSR) (Sato et al., 2012). Ghrelin and GHSR expression within the thymus decline with progressive age, and administration of both ghrelin and ghrelin‐receptor agonists alleviate age‐associated thymic deterioration in mice and humans (Dixit et al., 2007, 2009; Smith et al., 2007). Consistent with this, genetic studies revealed that ghrelin and GHSR deficiency accelerates age‐associated thymic demise in mice (Youm et al., 2009). Thus, many hormones contribute positively or negatively to thymic involution.

### 3.5 Adipocyte origin during thymic involution: The EMT process

Adipogenesis is a notable feature of thymic involution (Dixit, 2010). Recently, some progress has been made in understanding how adipocytes are formed during thymic aging. Using genetically modified reporter mice, Youm et al. first reported that TECs can transition to mesenchymal cells through a mechanism called EMT (Youm et al., 2009). These mesenchymal cells are highly plastic and have the potential to differentiate into adipocytes (Mani et al., 2008). Indeed, these mesenchymal cells express pro‐adipogenic genes, which provide a possible adipocyte origin in the thymus (Youm et al., 2009). Some regulators play critical roles in the EMT process. Peroxisome proliferator‐activated receptor gamma (PPARγ), a member of the nuclear receptor superfamily of ligand‐activated transcription factors, is involved in adipocyte development (Tontonoz & Spiegelman, 2008). Thus, it is reasonable to speculate that PPARγ may play a key role in thymic involution. Indeed, an adipocyte‐lineage‐specific constitutively active PPARγ transgene and administration of rosiglitazone, a PPARγ signaling activator, both promote age‐related thymic involution in mice (Youm et al., 2010). In fact, the Ghrl–GHSR interaction and CR both protect against thymic involution by inhibiting EMT and adipogenesis in mice (Yang, Youm, Vandanmagsar, et al., 2009; Youm et al., 2009), and CR‐mediated thymic involution inhibition also involves PPARγ downregulation in mouse thymic stromal cells (Yang, Youm, & Dixit, 2009). Furthermore, decreased Wnt4 and increased LAP2α during thymic aging may promote direct TEC trans‐differentiation into pre‐adipocytes or cause EMT and subsequent pre‐adipocyte differentiation (Kvell et al., 2010). A recent study showed that CD147 deletion from T cells in mice could prevent thymic involution by inhibiting TEC EMT, implying that the interaction between thymocytes and TECs contributes to age‐related thymic involution (Chen et al., 2020). Although some progress has been made, adipocyte origin during thymic aging needs further investigation.

## 4 CONCLUSIONS

Age‐related thymic involution contributes significantly to immunosenescence. Although some progress has been made in understanding the molecular regulation of thymic involution, the detailed molecular regulation network is still unclear. Comprehensive information about age‐related thymic involution is needed to promote thymic rejuvenation in the elderly. With advances in transcriptome analysis, significant progress has been made in understanding overall thymic stromal cell changes during aging, and the use of scRNA‐seq has revealed comprehensive TEC subset changes. Thymic aging is associated with the downregulation of cell cycle‐related genes and ribosome biogenesis‐related genes in TECs. Recent genetic studies have also identified some new thymic aging regulators, including FGF21, lamin‐B1, liver X receptors, and some miRNAs. With the current understanding of age‐related thymic involution, we can speculate that thymic stromal cells (especially TECs) offer potential targets for thymic rejuvenation in the elderly.

## AUTHOR CONTRIBUTIONS

Zhanfeng Liang wrote the manuscript; Xue Dong and Qian Zhang designed figures; Zhaoqi Zhang analyzed the RNA‐seq data; Yong Zhao reviewed the manuscript and supervised the work.

## CONFLICT OF INTEREST

All authors declare no conflicts of interest.

## Data Availability

Data sharing is not applicable to this article as no new data were created or analyzed in this study.
